# The Transcriptome of the Salivary Glands of *Amblyomma aureolatum* Reveals the Antimicrobial Peptide Microplusin as an Important Factor for the Tick Protection Against *Rickettsia rickettsii* Infection

**DOI:** 10.3389/fphys.2019.00529

**Published:** 2019-05-03

**Authors:** Larissa A. Martins, Camila D. Malossi, Maria F. B. de M. Galletti, José M. Ribeiro, André Fujita, Eliane Esteves, Francisco B. Costa, Marcelo B. Labruna, Sirlei Daffre, Andréa C. Fogaça

**Affiliations:** ^1^Departamento de Parasitologia, Instituto de Ciências Biomédicas, Universidade de São Paulo, São Paulo, Brazil; ^2^Laboratory of Malaria and Vector Research, National Institute of Allergy and Infectious Diseases, Bethesda, MD, United States; ^3^Departamento de Ciência da Computação, Instituto de Matemática e Estatística, Universidade de São Paulo, São Paulo, Brazil; ^4^Departamento de Imunologia, Instituto de Ciências Biomédicas, Universidade de São Paulo, São Paulo, Brazil; ^5^Departamento de Medicina Veterinária Preventiva e Saúde Animal, Faculdade de Medicina Veterinária e Zootecnia, Universidade de São Paulo, São Paulo, Brazil

**Keywords:** spotted fever, tick-rickettsiae interaction, immune, microplusin, antimicrobial peptide, salivary glands, transcriptome, RNAi

## Abstract

The salivary glands (SG) of ixodid ticks play a pivotal role in blood feeding, producing both the cement and the saliva. The cement is an adhesive substance that helps the attachment of the tick to the host skin, while the saliva contains a rich mixture of antihemostatic, anti-inflammatory, and immunomodulatory substances that allow ticks to properly acquire the blood meal. The tick saliva is also a vehicle used by several pathogens to be transmitted to the vertebrate host, including various bacterial species from the genus *Rickettsia*. *Rickettsia rickettsii* is a tick-borne obligate intracellular bacterium that causes the severe Rocky Mountain spotted fever. In Brazil, the dog yellow tick *Amblyomma aureolatum* is a vector of *R. rickettsii*. In the current study, the effects of an experimental infection with *R. rickettsii* on the global gene expression profile of *A. aureolatum* SG was determined by next-generation RNA sequencing. A total of 260 coding sequences (CDSs) were modulated by infection, among which 161 were upregulated and 99 were downregulated. Regarding CDSs in the immunity category, we highlight one sequence encoding one microplusin-like antimicrobial peptide (AMP) (Ambaur-69859). AMPs are important effectors of the arthropod immune system, which lack the adaptive response of the immune system of vertebrates. The expression of microplusin was confirmed to be significantly upregulated in the SG as well as in the midgut (MG) of infected *A. aureolatum* by a quantitative polymerase chain reaction preceded by reverse transcription. The knockdown of the microplusin expression by RNA interference caused a significant increase in the prevalence of infected ticks in relation to the control. In addition, a higher rickettsial load of one order of magnitude was recorded in both the MG and SG of ticks that received microplusin-specific dsRNA. No effect of microplusin knockdown was observed on the *R. rickettsii* transmission to rabbits. Moreover, no significant differences in tick engorgement and oviposition were recorded in ticks that received dsMicroplusin, demonstrating that microplusin knockdown has no effect on tick fitness. Further studies must be performed to determine the mechanism of action of this AMP against *R. rickettsii*.

## Introduction

Ticks are obligate blood feeding arthropods and their success in acquiring the host’s blood depends essentially on the physiological functions performed by the salivary glands (SG) (Bowman and Sauer, 2004; Sonenshine and Roe, 2013). Primarily, SG are osmoregulation organs that revert the excess of fluids and ions from the blood meal back into the host’s circulation via saliva. In this manner, the nutrients are concentrated and both the volume and the ionic composition of the hemolymph are regulated (Kaufman, 2010). The tick saliva also contains a myriad of biomolecules with diverse pharmacological activities, including anticoagulation, antiplatelet, vasodilatory, anti-inflammatory, and immunomodulatory, enabling blood uptake (Francischetti et al., 2009; Kazimirova and Stibraniova, 2013; Kotal et al., 2015; Simo et al., 2017). Infectious agents, including viruses, bacteria, and protozoa, use tick saliva as a vehicle to be transmitted to a vertebrate host (Dantas-Torres et al., 2012; Simo et al., 2017). In addition, the biological properties of the tick saliva were previously reported to benefit the transmission of viruses and bacteria to the host (Kaufman, 2010; Kazimirova and Stibraniova, 2013; Simo et al., 2017). Besides saliva, SG also produce the cement, an adhesive substance that covers the tick mouthparts and helps the attachment to the host skin, seals the feeding lesion, and prevents the contact of tick mouthparts with the host’s immune factors (Suppan et al., 2018).

*Rickettsia rickettsii* is a tick-borne obligate intracellular bacterium that causes the life-threatening Rocky Mountain spotted fever (RMSF). *R. rickettsii* colonizes the endothelial cells of the vertebrate host, causing an intense vasculitis that can lead to the failure of important organs, including the brain, lungs, and kidneys. Antibiotic treatment is available, but it is effective only if performed within a few days of illness onset (Chapman et al., 2006; Dantas-Torres, 2007; Chen and Sexton, 2008). Nonetheless, the non-specificity of clinical manifestations, such as fever, headache, and myalgia, associated with the late detection of antibodies to *R. rickettsii* in serological tests, make early diagnosis difficult (Dantas-Torres, 2007). As a consequence, fatality rates of the disease are still high, reaching approximately 40% in Brazil (Labruna, 2009). Specifically in the State of São Paulo, lethality rates can overpass 70% [official data from São Paulo State Health Secretary (2007–2018)].

In Brazil, *Amblyomma sculptum* [formerly named *Amblyomma cajennense* (Nava et al., 2014)] and *Amblyomma aureolatum* are implicated as vectors of *R. rickettsii* (Labruna, 2009). The tick midgut (MG) is the first tick organ that interacts with rickettsiae acquired within the blood meal. The rickettsiae then need to reach the SG to be transmitted to the vertebrate host via saliva. Importantly, rickettsiae are not only collected from the hemolymph by the tick SG, but actively proliferate in this organ (Socolovschi et al., 2009). We previously showed that the infection with *R. rickettsii* modulates the global gene expression profile of the MG of both *A. sculptum* and *A. aureolatum* (Martins et al., 2017). The majority of modulated coding sequences (CDSs) of *A. aureolatum*, which is more susceptible to the *R. rickettsii* infection than *A. sculptum* (Labruna et al., 2008), were downregulated in response to infection (Martins et al., 2017). On the other hand, most *A. sculptum* CDSs, including immune factors, were upregulated in the MG of infected ticks. In the current study, we determined the global transcriptional profile of *A. aureolatum* SG in response to an infection with *R. rickettsii* by next-generation RNA sequencing (RNA-seq). Ticks were infected by feeding on infected hosts, mimicking a natural infection. RNA-seq data were validated by a quantitative polymerase chain reaction preceded by reverse transcription (RT-qPCR). The coding sequence (CDS) of one antimicrobial peptide with similarity to the microplusin of *Rhipicephalus microplus* (Fogaca et al., 2004), which was significantly induced by infection, was targeted for functional characterization using RNA interference (RNAi). Besides generating a transcript databank of *A. aureolatum* SG, our data showed that microplusin is one important factor of tick-rickettsiae interactions.

## Materials and Methods

### Ethics Statement

The procedures adopted for the experiments involving vertebrate animals were approved by the Institutional Animal Care and Use Committees from the Faculty of Veterinary Medicine (protocol number 1423/2008) and the Institute of Biomedical Sciences (protocol number 128/2011), University of São Paulo, São Paulo, Brazil.

### *R. rickettsii*-Infected and Uninfected Ticks

Adult ticks, infected or not with the highly virulent Taiaçu strain of *R. rickettsii*, were obtained using the procedure previously detailed in Pinter and Labruna (2006) and Galletti et al. (2013). Off-host phases were held in an incubator at 25°C and 95% relative humidity. The SG and MG of each tick were dissected and separately transferred to 100 μL of RNA*later*^®^ Solution (Thermo Fisher Scientific, United States).

### Nucleic Acid Extraction

The SG and MG of each adult tick were homogenized and submitted to a simultaneous isolation of genomic DNA (gDNA) and total RNA using the InviTrap^®^ Spin Cell RNA Mini Kit (Stratatec, Germany) according to the manufacturer’s specifications.

### Real-Time Quantitative PCR (qPCR) to *R. rickettsii* Quantification

Genomic DNA was utilized as a template to quantify the total number of rickettsiae in tick organs by real-time quantitative PCR (qPCR) using a hydrolysis probe for the citrate synthase gene (*glt*A) of *R. rickettsii*, as previously described (Galletti et al., 2013). gDNA samples extracted from non-infected ticks were also analyzed to confirm the absence of infection. All samples were analyzed in three technical replicates.

### RNA-Seq, Assembly, and Annotation

The RNA extracted from the SG of ten *A. aureolatum* harboring between 7.00 × 10^4^ and 1.00 × 10^5^ rickettsiae was pooled to generate the infected sample. The RNA extracted from the SG of ten non-infected *A. aureolatum* was also combined to generate the control sample. Each tick contributed equally for the composition of the two pool samples, which were submitted to a high throughput mRNA sequencing (RNA-seq), together with RNA samples from the MG of non-infected and infected *A. aureolatum* (Martins et al., 2017) and from the SG of fed and unfed *A. sculptum* (Esteves et al., 2017). To that end, all samples were tagged and multiplex sequenced in four lanes using a HiSeq^TM^ sequencing system (Illumina, United States) at the North Carolina State University facility (NC, United States). A total of around 242 million reads of 101 base pairs were obtained for *A. aureolatum* samples using the single read and submitted to a bioinformatics analysis as detailed before (Karim et al., 2011; Esteves et al., 2017; Martins et al., 2017).

Paired comparisons of the number of reads hitting each CDS were calculated by chi-squared test to detect significant differences between the gene expression in SG of *A. aureolatum* infected (AaI) and non-infected (AaC). Normalized fold-ratios of the sample reads were computed by adjusting the numerator by a factor based on the ratio of the total number of reads in each sample and adding one to the denominator to avoid division by zero. The minimum considered fold-change in infected ticks in relation to control ticks was larger than five and *p* < 0.05.

The complete dataset was organized in a hyperlinked spreadsheet as previously reported (Ribeiro et al., 2004) and a table with links (Supplementary Table 1) may be downloaded from https://s3.amazonaws.com/proj-bip-prod-publicread/transcriptome/Amb_aureolatum/SupplementaryTable1.zip. The raw data were deposited to the Sequence Read Archives (SRA) of the NCBI under the BioProject PRJNA344771 [raw reads runs SRR4301100 (SG of control *A. aureolatum*) and SRR4301110 (SG of infected *A. aureolatum*)]. The Transcriptome Shotgun Assembly (TSA) project has been deposited at DDBJ/EMBL/GenBank under the accession code GFAC00000000. Only CDSs representing 90% of the sequences of known proteins or larger than 250 amino acids were deposited.

The amino acid sequence of the protein encoded by the CDS Ambaur-69859 (GenBank protein ID: JAT93257.1), whose code one AMP was similar to the microplusin of *R. microplus* (Fogaca et al., 2004), was used as query in blastp searches against both Transcriptome Shotgun Assembly (tsa_nr; NCBI) and UniProtKB/Swiss-Prot (swissprot) databases with the phylum Arthropoda (taxid: 6656) as filter. The protein sequence of a given tick species with the best match with *A. aureolatum* was selected as representative for that species and used to perform multiple sequence alignment (MSA) using the multiple sequence comparison by log-expectation (MUSCLE) tool (Edgar, 2004a,b) with default parameters at the European Bioinformatics Institute (EMBL-EBI) website (McWilliam et al., 2013; Li et al., 2015). A phylogenetics analysis of microplusins (Supplementary Table 2) was performed with the Maximum Likelihood (ML) method with the Jones-Taylor-Thornton (JTT) matrix-based substitution model (Jones et al., 1992) using MEGA X (Kumar et al., 2018) software.

### Validation of RNA-Seq Data Using Real-Time Quantitative PCR Proceeded by Reverse Transcription (RT-qPCR)

Five hundred nanogram of the total RNA extracted from the SG of non-infected (AaC) or infected (AaI) ticks were treated with RQ1 RNase-free DNase (Promega, United States) and reverse transcribed (RT) into cDNA using M-MLV Reverse Transcriptase (Thermo Fisher Scientific, United States), as detailed by the manufacturer. The resulting cDNA was used as a template in qPCR with the Maxima SYBR Green/ROX qPCR MasterMix (Thermo Fisher Scientific, United States) and specific primers for selected CDSs (Supplementary Table 3). Primers were designed using Primer3 (Rozen and Skaletsky, 2000) and synthesized by Thermo Fisher Scientific (United States). qPCR was performed on a StepOne Plus thermocycler (Thermo Fisher Scientific, United States) using the following program: 95°C for 10 min followed by 40 cycles at 95°C for 15 s, 60°C for 60 s, and 72°C for 20 s. The 2^-ΔΔCt^ equation was utilized to calculate the relative expression of select genes in infected versus non-infected ticks or exposed versus infected ticks (Livak and Schmittgen, 2001). The encoding gene of the ribosomal protein S3A was used as reference (Martins et al., 2017). At least three biological replicates of each group were analyzed. Student’s *t*-test was used to statistically validate the differentially expressed CDSs.

### RNA Interference in Ticks

The knockdown of microplusin (CDS Ambaur-69859) was induced by injection of double-stranded RNA (dsRNA) into adult tick hemocoel as described by Kocan et al. (2011). Briefly, specific primers containing T7 promoter sequence (Supplementary Table 3) were used to amplify the target sequence (Ambaur-69859) by PCR. The non-related dsRNA of the merozoite surface protein 1 (MSP1) of *Plasmodium falciparum* was used as control (Supplementary Table 3; Kalil et al., 2017). The resulting products were purified using PCR Purification GeneJet^TM^ kit (Thermo Fisher Scientific, United States). One microgram of the purified cDNA was utilized to synthesize dsRNAs using the T7 Ribomax Express RNAi System kit (Promega, United States).

To evaluate the effects of gene silencing on the acquisition of *R. rickettsii*, non-infected adult ticks were injected with 10^11^ molecules of either dsMicroplusin (333 base pairs) or dsMSP1 (666 base pairs) in 69 nL of PBS (12 ticks in each group). An injection was administered in the coxal membrane located at the base of the fourth leg using Nanoject II equipment (Drummond). After injection, ticks were kept in an incubator at 25°C and 95% RH for 24 h. Ticks were fed on infected rabbits during the bacteremia peak. After 3 days, ticks were manually removed, and SG and MG were dissected as described above.

To evaluate the effects of silencing on the transmission of *R. rickettsii*, adult ticks infected during feeding of the larval stage were injected with specific dsRNA using the same equipment and procedure described above (60 ticks in each group). Twenty-four hours after dsRNA administration, ticks were allowed to feed on non-infected rabbits (30 ticks per rabbit). After 5 days, six ticks from each group were manually removed and SG were dissected. The rectal temperature of rabbits was monitored daily and fever was considered when the temperature was higher than 40°C. Skin biopsies of the rabbits were performed on days 3, 10, and 21 after the beginning of tick feeding using 3 mm punches under local anesthesia. In addition, blood samples of all rabbits were collected on days 0 and 21 postinfestation for immunofluorescence assays (IFA), as detailed by Horta et al. (2008).

To determine the effects of gene silencing on *A. aureolatum* fitness, non-infected adults were injected with either dsMicroplusin or dsMSP1. Ticks fed on one non-infected dog until they were completely engorged. Females were individually weighted and transferred to an incubator at 25°C and 95% RH. After the end of oviposition, the egg mass of each female was weighted. The fertility rate was obtained by calculating the ratio between the weight of the egg mass and the weight of each female.

To evaluate gene silencing, total RNA extracted from tick organs was used as a template in RT-qPCR following the same procedure described above. To calculate the percentage of gene silencing in ticks injected with dsMicroplusin, we considered the levels of their respective transcripts in the control group as 100%. The gDNA extracted from tick organs and from skin biopsies of rabbits was used as a template in qPCR for *R. rickettsii* quantification, as described above. Differences in the microplusin gene expression, rickettsial load, and tick fitness parameters were analyzed between dsMicroplusin and dsMSP1 groups by Mann–Whitney test using GraphPad Prism version 7.0 for Windows (GraphPad Software, United States) and considered significant when *p* < 0.05.

## Results

### Identification of Differentially Expressed CDSs in SG of *A. aureolatum* Ticks in Response to the *R. rickettsii* Infection

Approximately 242 million reads were obtained from the analysis of RNA samples extracted from *A. aureolatum* organs by RNA-seq, among which nearly 110 million reads corresponded to SG samples [number of reads in SG infected (AaI): 50,475,296; number of reads in SG control (AaC): 59,846,982]. The total of 242 million reads was assembled into 11,906 CDSs from which 11,903 are expressed in SG and 11,888 in MG (Supplementary Table 1).

**FIGURE 1:**
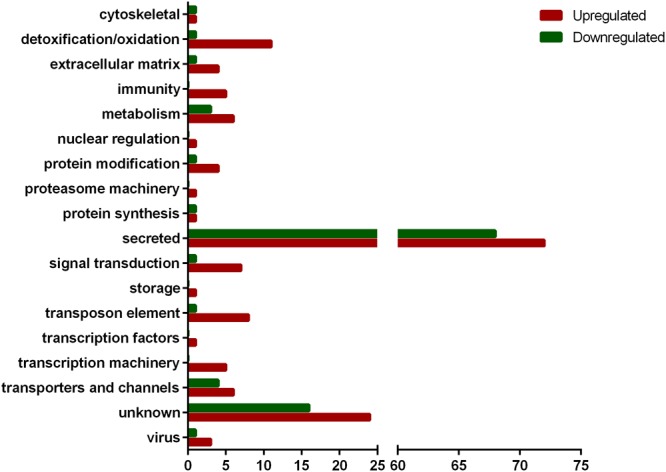
Functional classification of CDSs differentially expressed in SG of *R. rickettsii*-infected *A. aureolatum* ticks.

From the 11,903 CDSs expressed in SG, 161 were upregulated by infection while 99 were downregulated (Supplementary Table 1 and Table 1). The majority of modulated CDSs encode putative secreted proteins (72 upregulated and 68 downregulated) (Figure 1), including lipocalins and Kunitz-type inhibitors. Twelve CDSs of putative secreted lipocalins were downregulated by infection, while only six were upregulated. Regarding CDSs encoding putative secreted Kunitz-type inhibitors, seven were downregulated and only one was upregulated by infection (Supplementary Table 1). The functional classes detoxification/oxidation, transposon elements, signal transduction, protein modification, transporter and channels, metabolism, and extracellular matrix comprise CDSs mostly upregulated by infection (Figure 1). In the detoxification/oxidation category, seven CDSs encoding cytochrome P450 (CYP) were upregulated in the SG of infected ticks, while only one was downregulated (Supplementary Table 1, Table 1, and Figure 1). In addition, two glutathione *S*-transferase (GST) CDSs were upregulated in response to infection and none was downregulated (Supplementary Table 1 and Table 1).

**Table 1:** Selected CDSs of *A. aureolatum* salivary glands differentially expressed by infection with *R. rickettsii*.

CDS	Annotation	Functional class	Fold-change	
			RNA-seq	RT-qPCR
Ambaur-1956	Cytochrome P450	Detoxification/oxidation	9.16	NA
Ambaur-60624	Cytochrome P450		5.02	NA
Ambaur-67466	Cytochrome P450		5.32	NA
Ambaur-45304	Cytochrome P450		5.48	NA
Ambaur-36571	Cytochrome P450		9.12	NA
AmbarSigP-56564	Cytochrome P450		8.01	NA
Ambaur-60175	Cytochrome P450		11.74	2.27^∗^
Ambaur-61062	Cytochrome P450		0.17	NA
Ambaur-47534	Glutathione *S*-transferase		5.16	NA
Ambaur-23052	Glutathione *S*-transferase		7.49	NA
Ambaur-18924	Peptidoglycan recognition protein	Immunity	5.81	NA
AmbarSigP-61895	α-Macroglobulin		22.12	NA
AmbarSigP-5190	α-Macroglobulin		17.87	0.65
Ambaur-69859	Microplusin		8.52	4.71^∗^
Ambaur-25218	Microplusin		5.93	NA
AmbarSigP-22173	Microplusin	Secreted	0.10	0.44^∗^
Ambaur-50152	5.3 kDa antibacterial peptide		0.00	NA
Ambaur-19862	5.3 kDa antibacterial peptide		0.16	0.005
AmbarSigP-47654	Lipocalin		37.90	3.12
Ambaur-47378	Basic tail protein		6.42	133,707.32^∗∗^
AmbarSigP-70152	Basic tail protein		0.02	0.05^∗∗^
Ambaur-6577	8.9 kDa protein		0.05	0.18
Ambaur-54200	Secreted protein		0.13	141.58^∗^
Ambaur-4333	Salivary secreted protein of 21.3 kDa	Unknown conserved	11.46	0.79
Ambaur-22275	Polyubiquitin	Proteasome machinery	5.84	NA
Ambaur-53071	Histone 2B	Nuclear regulation	6.44	NA

*Only CDSs with statistically significant differences in expression in the salivary glands of infected vs. control (non-infected) ticks, identified by RNA-seq analysis, are presented. Some CDSs were additionally analyzed by RT-qPCR (^∗^*p* < 0.05 and ^∗∗^*p* < 0.01; Student’s *t*-test). NA, relative expression not analyzed by RT-qPCR.*

The functional categories of nuclear regulation, proteasome machinery, storage, and transcription factors are represented exclusively by upregulated CDSs and each of them is composed of only one CDS (Figure 1). Among them, we highlight one polyubiquitin (Ambaur-22275) and one histone 2B (Ambaur-53071) encoding sequences, included in proteasome machinery and nuclear regulation categories, respectively. In the immunity category, five CDSs were upregulated by infection: one peptidoglycan receptor protein (PGRP; Ambaur-18924), two α-macroglobulins (AmbarSigP-61895 and AmbarSigP-5190), and two antimicrobial peptides (AMPs) similar to the microplusin of *R. microplus* (Fogaca et al., 2004) (Ambaur-69859 and Ambaur-25218) (Table 1). Moreover, one CDS of a trypsin-inhibitor like (TIL) (Ambaur-46428), a type of protease inhibitor previously reported to exhibit antimicrobial activity (Fogaca et al., 2006; Wang et al., 2015), was also upregulated by *R. rickettsii*. Conversely, one microplusin (AmbarSigP-22173) and two 5.3 kDa antimicrobial peptides (Ambaur-50152 and Ambaur-19862), were downregulated in infected ticks in relation to the control (Supplementary Table 1 and Table 1).

The expression of 11 CDSs was further analyzed by RT-qPCR (Table 1). Five of them [two microplusins (Ambaur-69859 and AmbarSigP-22173), one cytochrome P450 (Ambaur-60175), and two basic tail proteins (Ambaur-47378 and AmbarSigP-70152)] were significantly modulated in infected versus control ticks, exhibiting the same transcriptional profile observed in RNA-seq. Three additional CDSs [one 5.3 kDa antimicrobial peptide (Ambaur-19862), one lipocalin (AmbarSigP-47654) and one 8.9 kDa protein (Ambaur-6577)] presented the same expression pattern previously obtained by RNA-seq, but their expression levels in infected ticks were not significantly different in relation to control ticks. Likewise, the transcription of the CDSs Ambaur-4333 and AmbarSigP-5190, which encode a salivary secreted protein of 21.3 kDa and an α-macroglobulin, respectively, was not significantly different in infected versus control ticks. One CDS of a putative secreted protein (Ambaur-54200) presented the opposite pattern obtained by RNA-seq, detected as significantly upregulated in infected ticks by RT-qPCR.

### Microplusin Functional Study on *R. rickettsii* Acquisition and Transmission

**FIGURE 2:**
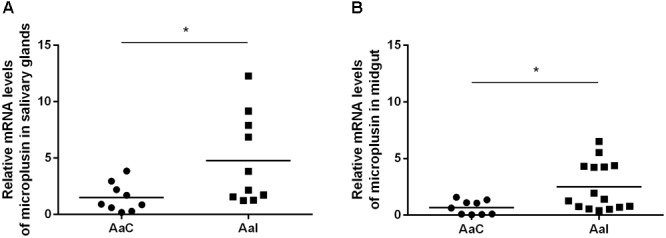
Relative expression of microplusin in salivary glands **(A)** and midgut **(B)** of ticks infected (AaI) or not (control; AaC) with *R. rickettsii* by RT-qPCR (^∗^*p* < 0.05; Mann–Whitney test).

**FIGURE 3:**
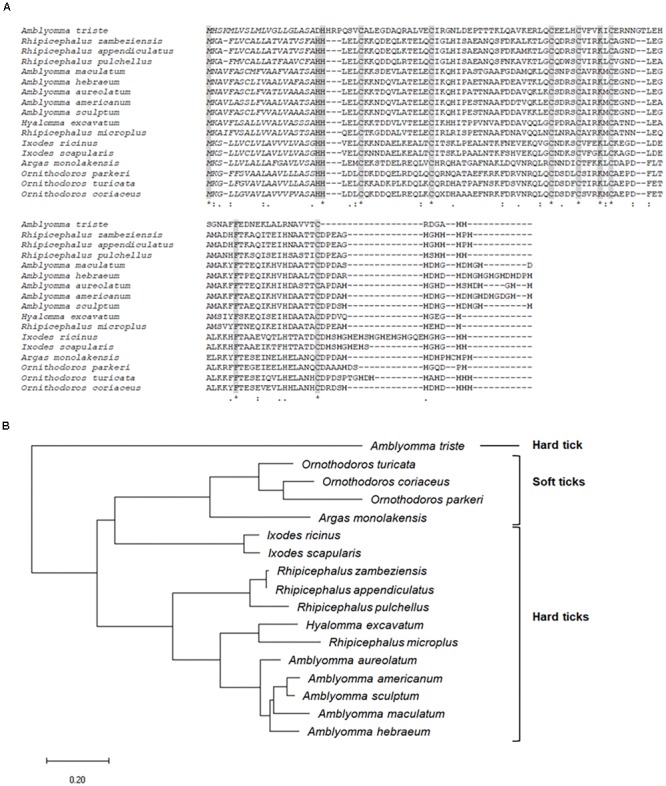
MSA and phylogenetic analysis of microplusins. **(A)** Multiple sequence alignment of protein sequences was performed using MUSCLE method. Asterisks highlight the conserved cysteine residues; italic letters show the signal peptide; bold letters indicate histidine residues in the C-terminal. **(B)** A phylogenetic tree was constructed with protein sequences using Maximum Likelihood (ML) method. The analysis involved 17 amino acid sequences (accession numbers available in Supplementary Table 2). Bar scale at the bottom indicates 20% amino acid divergence.

Besides being significantly upregulated by infection in SG of infected ticks, the CDS Ambaur-69859, which encodes the antimicrobial peptide microplusin, was also upregulated in MG (Figure 2). The MSA analysis of the amino acid sequence of *A. aureolatum* microplusin with microplusins of different species of soft and hard ticks showed that all sequences present a conserved signal peptide as well as six conserved cysteine residues (Figure 3A). The mature peptide of all sequences starts with two histidine residues, except for *Amblyomma triste*, which sequence starts with an aspartate residue followed by two histidine residues. All of them also exhibit a histidine-rich C-terminal with a variable number of histidine residues after the last cysteine residue, ranging from two (*A. triste* and *Hyalomma excavatum*) to 12 (*Ixodes ricinus*). The phylogenetic tree showed that all species in the genus *Amblyomma* cluster together, with the exception of *A. triste* (Figure 3B). In fact, this last species is the most distant sequence in relation to the *Amblyomma* cluster and lays with soft ticks (genera *Argas* and *Ornithodoros*). The hard ticks *R. microplus* and *H. excavatum* cluster together and with other species in the genus *Rhipicephalus*. Microplusins of the two analyzed species of the genus *Ixodes* (*Ixodes ricinus* and *Ixodes scapularis*) also cluster together and are close to soft ticks.

**FIGURE 4:**
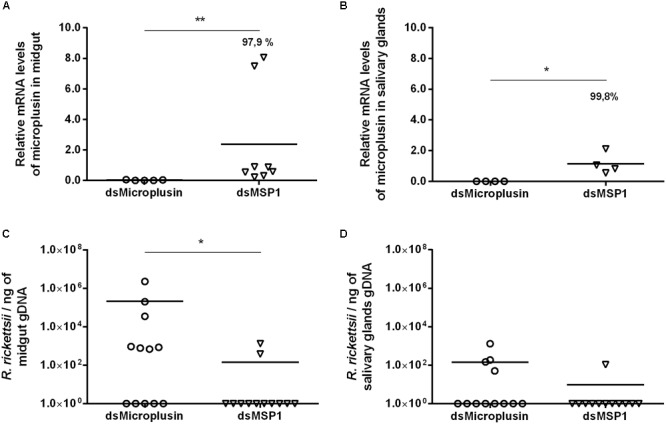
Effect of RNAi-mediated silencing of microplusin in *R. rickettsii* acquisition by *A. aureolatum*. Specific dsRNA for either microplusin (dsMicroplusin) or MSP1 (dsMSP1; control) were injected into the hemocoel of non-infected ticks. After 24 h, ticks were fed on *R. rickettsii*-infected rabbits for 3 days. The relative expression of microplusin in midgut **(A)** and salivary glands **(B)** of ticks injected with dsMicroplusin in relation to the control was assessed by RT-qPCR and the rickettsial load in MG **(C)** and SG **(D)** by qPCR (^∗^*p* < 0.05 and ^∗∗^*p* < 0.005; Mann–Whitney test).

The CDS Ambaur-69859 was selected for the functional studies assessed by RNAi experiments. Specific dsRNA for either microplusin or MSP1 (control) were injected into the hemocoel of *A. aureolatum*. A survival rate of 100% was obtained 24 h post-injection and ticks were then fed on infected rabbits. A high efficiency of microplusin silencing of around 97.9% in the MG (Figure 4A) and 99.8% in the SG (Figure 4B) was obtained. A higher prevalence of ticks with infected MG was obtained in the group of ticks that received dsMicroplusin (58.3%) in comparison with the control group (16.7%). The prevalence of ticks with infected SG was also higher in the dsMicroplusin group (33.3%) than in the dsMSP1 group (8.3%). In addition, the rickettsial load of one order of magnitude was higher in the MG of ticks that received dsMicroplusin, with significant differences in comparison to the control (Figure 4C). The rickettsial load was also higher in SG of ticks from dsMicroplusin group, although not significant (Figure 4D).

**FIGURE 5:**
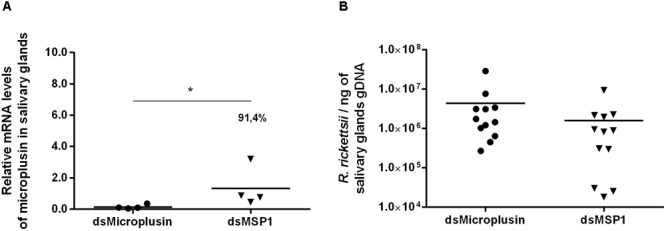
Relative expression of microplusin and rickettsial load in ticks used to evaluate the effects of gene-knockdown on *R. rickettsii* transmission to rabbits. Specific dsRNA for either microplusin (dsMicroplusin) or MSP1 (dsMSP1; control) were injected into the hemocoel of *R. rickettsii*-infected ticks. After 24 h, ticks were fed on non-infected rabbits for 5 days. The relative expression of microplusin **(A)** and the rickettsial load **(B)** in SG of ticks injected with dsMicroplusin in relation to the control were assessed by RT-qPCR and by qPCR, respectively (^∗^*p* < 0.05; Mann–Whitney test).

The effects of microplusin knockdown on the transmission of *R. rickettsii* to rabbits were also evaluated. To that end, *R. rickettsii*-infected ticks were injected with either dsMicroplusin or dsMSP1. Despite the fact that the microplusin expression had been significantly silenced in the group that received dsMicroplusin in relation to the control group (Figure 5A), no difference was identified between both groups. All rabbits exhibited fever, were *R. rickettsii*-positive in skin biopsies and/or died during the course of infestation, independently on the tick group that they were exposed to (Table 2). The rickettsial load in the SG of ticks removed from the rabbits 5 days after the beginning of feeding was similar in both groups (Figure 5B).

**Table 2:** Effect of RNAi-mediated silencing of microplusin in *R. rickettsii* transmission to rabbits.

Tick group	Rabbit	Fever (>40°C)	Skin biopsy positive in qPCR	Death	IFA
dsMicroplusin	1	On days 7–8th	1.33 × 10^2^	On day 11th	NA
	2	On days 7–10th	–	–	+
dsMSP1	3	On days 7–12th	–	–	+
	4	On days 8–9th	3.18 × 10^2^	On day 13th	NA

*Specific dsRNA for either microplusin (dsMicroplusin) or MSP1 (dsMSP1; control) were injected into the hemocoel of infected ticks. After 24 h, ticks were fed on non-infected rabbits. The temperature of all animals was daily evaluated, and it was considered fever when >40°C. gDNA extracted from skin biopsies of rabbits was used as template in qPCR for *R. rickettsii* detection. Blood samples of survive rabbits were collected on day 21 postinfestation and tested for anti-*R. rickettsii* reactive antibodies by IFA. NA, not analyzed.*

**FIGURE 6:**
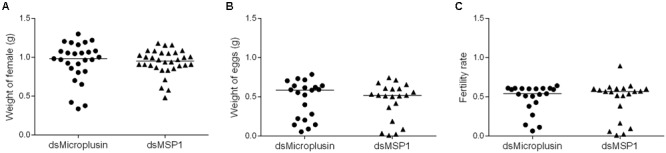
Effects of RNAi-mediated silencing of microplusin on tick fitness. Specific dsRNA for either microplusin (dsMicroplusin) or MSP1 (dsMSP1; control) were injected into the hemocoel of non-infected ticks. After 24 h, ticks were fed on one non-infected dog. The weight of engorged ticks **(A)**, weight of the egg mass **(B)**, and fertility rate **(C)** were recorded.

To evaluate the effect of microplusin knockdown on the fitness of *A. aureolatum* adult females (Figure 6), engorged females were weighted (Figure 6A) as well as their laid eggs (Figure 6B) and used to calculate their fertility rate (Figure 6C). No significant difference was detected among all fitness parameters in microplusin silenced ticks when compared to the control group.

## Discussion

Pathogens acquired by ticks within the blood meal have to overcome several barriers within ticks, including the MG, hemolymph, and SG, to be efficiently transmitted to another host. Each of these organs plays a decisive role in the tick vector competence for a particular pathogen (Ueti et al., 2007; Hajdušek et al., 2013). We have previously reported the effects of *R. rickettsii* on the global gene expression profile of the MG of *A. aureolatum* and *A. sculptum* (Martins et al., 2017). In the current study, we report the transcriptional profile of the SG of *A. aureolatum* in response to infection with this same pathogen. To date, the availability of, albeit partial, genomes of ticks is restricted to *I. scapularis* (Gulia-Nuss et al., 2016). Therefore, this transcriptomic study provides a rich source for tick sequences in the genus *Amblyomma*.

Around 242 million reads obtained by RNA-seq of RNA samples extracted from organs of *A. aureolatum* were assembled into 11,906 CDSs. Transcripts of the majority of assembled CDSs were detected with at least one read in tick SG, among which 21.2% encode proteins with a signal peptide for secretion. This result is in accordance with the secretory role played by tick SG (Bowman and Sauer, 2004; Kaufman, 2010). CDSs of putative secreted proteins count for 53.8% of the total of sequences modulated by *R. rickettsii* infection. From those, we highlight CDSs of lipocalins and Kunitz inhibitors, which were mostly downregulated by infection. Lipocalins are proteins of tick saliva that possess the property of binding the inflammatory mediators histamine and serotonin and, therefore, they play an anti-inflammatory role (Paesen et al., 1999; Sangamnatdej et al., 2002). A tendency of higher *Babesia bovis* infection, despite not significant, was observed in the larval progeny of lipocalin-silenced *R. microplus* females (Bastos et al., 2009). Besides downregulation in SG, our previous study showed that most CDSs of lipocalins were also downregulated in the MG of *A. aureolatum* in response to *R. rickettsii* infection. In contrast to the *A. aureolatum* pattern, all modulated CDSs of lipocalins were upregulated in the MG of infected *A. sculptum* (Martins et al., 2017). Therefore, it is possible that inflammatory components acquired within the blood meal exert a detrimental effect on the MG epithelium, enabling *A. aureolatum* to be more susceptible to infection than *A. sculptum*.

Most of the CDSs of Kunitz inhibitors modulated by infection were downregulated in the SG of *A. aureolatum*, as previously described for the tick MG (Martins et al., 2017). Conversely, all modulated CDSs of Kunitz inhibitors were upregulated in the MG of infected *A. sculptum* (Martins et al., 2017). It is known that Kunitz inhibitors of tick saliva exert an inhibitory activity on blood coagulation, assuring the acquisition of a fluid blood meal (Corral-Rodriguez et al., 2009; Francischetti et al., 2009). Moreover, it was previously reported that a Kunitz inhibitor of *Dermacentor variabilis* possesses a bacteriostatic effect against the non-virulent *Rickettsia montanensis* (Ceraul et al., 2008) and that its knockdown increases tick infection (Ceraul et al., 2011). Therefore, it is plausible to suppose that Kunitz inhibitors may also exhibit an antimicrobial activity against *R. rickettsii*, controlling infection in the MG of *A. sculptum* and culminating in a low susceptibility to infection.

Considering upregulated CDSs identified in the current study, some are included in the detoxification/oxidation category and code CYPs and GSTs. CYPs constitute an ancient protein family ubiquitous in nature and have been described to play different roles, being involved in pesticide detoxification as well as in the synthesis of different important endogenous molecules, such as hormones (Werck-Reichhart and Feyereisen, 2000). Sixty-eight CDSs of CYPs were detected in the *A. aureolatum* transcriptome, among which seven were upregulated and only one was downregulated in the SG of infected ticks. The upregulation of CYP genes in response to infection was previously reported, for instance in the mosquito *Aedes aegypti* (Behura et al., 2011) and in the honeybee *Apis mellifera* (Cornman et al., 2013). Intriguingly, the knockdown of CYP by RNAi reduced the infection of the mosquito *Anopheles gambiae* by *Plasmodium berghei* (Felix and Silveira, 2011). GSTs, which are involved in the detoxification of xenobiotics and oxidative stress (Pavlidi et al., 2018), were also previously reported to be upregulated by infection in arthropods, such as ticks (Mulenga et al., 2003; Mercado-Curiel et al., 2011; Bifano et al., 2014). For instance, one CDS of glutathione *S*-transferase was upregulated in the cell line BME26 of *R. microplus* and also in both MG and SG adult males by infection with *Anaplasma marginale*, the causative agent of bovine anaplasmosis. However, GST knockdown induced by RNAi presented no effect in either the acquisition of *A. marginale* by ticks or transmission to calves (Bifano et al., 2014). On the other hand, GST-knockdown reduced the acquisition of *A. marginale* by the tick *D. variabilis*, suggesting that the susceptibility response obtained by GST-silencing depends on the tick species and/or the pathogen strain (Kocan et al., 2009).

One polyubiquitin (Ambaur-22275), included in proteasome machinery category, was also upregulated in the SG of infected *A. aureolatum*. The upregulation of a polyubiquitin was previously reported to occur in the cell line IDE8 of *I. scapularis* in response to the infection of *A. marginale* (de la Fuente et al., 2007). The ubiquitination of proteins is a well-known signal for the degradation of protein in the proteasome, but it is also involved in the signaling of many other cellular processes (Welchman et al., 2005). Interestingly, the knockdown of the E3 [named x-linked inhibitor of apoptosis protein (XIAP)] of *I. scapularis* by RNAi restricted the proliferation of *Anaplasma phagocytophilum*, the causative agent of human granulocytic anaplasmosis (Severo et al., 2013).

The transcriptional level of one sequence coding for a histone 2B (Ambaur-53071), included in nuclear regulation, was also higher in the SG of infected *A. aureolatum*. The upregulation of histone 2B expression was already observed in the SG and MG of the tick *I. scapularis* infected with *A. phagocytophilum* (Cabezas-Cruz et al., 2016). The knockdown of the histone 2B of *I. scapularis* by RNAi was reported to impair the invasion of the cell line ISE6 by *Rickettsia felis*, an agent of flea-borne spotted fever. Moreover, it was shown that histone 2B interacts with the outer membrane protein B of *R. felis*, suggesting that it might play a role in cell invasion (Thepparit et al., 2010).

Some immunity components were also differentially expressed in the SG of infected *A. aureolatum*. Among them, one sequence encoding PGRP (Ambaur-18924) was upregulated. PGRPs are classified into non-catalytic or catalytic depending on the presence of an amidase catalytic site. The non-catalytic PGRPs act as pathogen pattern recognition receptors and activate the Toll and Imd pathways, as demonstrated in *Drosophila*. On the other hand, catalytic PGRPs cleave peptidoglycan, acting as either effectors, by exerting an antibacterial activity, or negative regulators of the immune response, by performing the clearance of peptidoglycan (Palmer and Jiggins, 2015). As the PGRP putatively coded by CDS Ambaur-18924 exhibits the amidase catalytic site (data not shown), it may play a role as an effector or negative regulator of tick immune signaling pathways. Two α-macroglobulins (AmbarSigP-61895 and AmbarSigP-5190) were also upregulated by infection. Alpha-macroglobulins are members of the family of thioester-containing proteins (TEPs) (Buresova et al., 2011; Urbanova et al., 2015). Interestingly, the knockdown of α2-macroglobulins mediated by RNAi diminished the phagocytosis of *Chryseobacterium indologenes* by the hemocytes of *I. ricinus* (Buresova et al., 2011). Additionally, the transcriptional levels of one trypsin-inhibitor like (TIL) encoding sequence (Ambaur-46428), a type of protease inhibitors that exhibit antimicrobial activity (Fogaca et al., 2006; Wang et al., 2015), was also higher in infected *A. aureolatum* ticks.

The immune system of invertebrates, including arthropods, is simpler than the immune system of vertebrates, lacking the adaptive response (Baxter et al., 2017). In this context, effector molecules, such as the aforementioned factors and AMPs, play a central role, acting directly against pathogens (Bulet et al., 2004). Interestingly, it was previously reported that the AMP defensin-2 reduces the *R. montanensis* load in the MG of the tick *D. variabilis* (Pelc et al., 2014). In addition, it was shown that this AMP causes the death of *R. montanensis* through lysis and leakage of cytoplasmic proteins (Pelc et al., 2014). Two microplusin CDSs (Ambaur-69859 and Ambaur-25218) were upregulated in the SG of *A. aureolatum* ticks by *R. rickettsii*. Besides being significantly upregulated by infection in the SG of infected ticks, the CDS Ambaur-69859 was also detected to be upregulated in MG. Microplusin is a 10,204 kDa AMP that was originally identified in the hemolymph (Fogaca et al., 2004) and lately in the ovary and eggs (Esteves et al., 2009) of *R. microplus*. Interestingly, this AMP is also upregulated in the SG and MG of *A. marginale*-infected *R. microplus* (Capelli-Peixoto et al., 2017). We therefore targeted the microplusin encoded by the CDS Ambaur-69859 to analyze the effects of its knockdown in the acquisition of the bacterium *R. rickettsii* by *A. aureolatum*. A higher prevalence of infected ticks was obtained in the dsMicroplusin injected group than in the control group, showing that microplusin silencing benefited *R. rickettsii* establishment within the tick. Moreover, the rickettsial load was also higher in both SG and MG of ticks that received dsMicroplusin, demonstrating that this AMP is important for the control of infection in both organs. On the other hand, microplusin knockdown showed no detectable effect on the transmission of *R. rickettsii* to rabbits. All together, these results show that microplusin plays a protective role against tick infection by *R. rickettsii* but does not exert any detectable effect on the bacterial transmission to rabbits after SG had already been infected by this bacterium. In addition, ticks that received either dsMicroplusin or dsMSP1 showed no significant differences in engorgement and oviposition, showing that the silencing of this AMP presents no effect on tick fitness.

The MSA analysis of the amino acid sequence of *A. aureolatum* microplusin with the amino acid sequences of microplusins of different species of ticks showed all of them exhibit six conserved cysteine residues and a histidine-rich C-terminal. Interestingly, the histidine-rich C-terminal of the microplusins of *R. microplus* (Silva et al., 2009, 2011) and *A. hebraeum* (Lai et al., 2004), which is named hebraein, was enrolled with their antimicrobial activity. Indeed, the histidine residues of the microplusin of *R. microplus* have the property of chelating metallic ions, such as copper, affecting the respiration of the Gram-positive bacterium *Micrococcus luteus* (Silva et al., 2009) and the fungus *Cryptococcus neoformans* (Silva et al., 2011). In addition, the copper-binding property of microplusin also alters the melanization and formation of the polysaccharide capsule of *C. neoformans* (Silva et al., 2011). Therefore, it would be interesting to determine the mechanism of action of microplusin against *R. rickettsii*.

## Conclusion

In conclusion, our data show that *R. rickettsii* exerts a modulatory effect on the transcriptional profile the SG of *A. aureolatum*. Moreover, RNAi experiments demonstrated that the knockdown of one microplusin increases the susceptibility of ticks to infection, suggesting that this is one important factor for the control of *R. rickettsii*. The functional characterization of the additional CDSs modulated by infection is warranted and might reveal other factors that interfere with the acquisition and/or transmission of this tick-borne pathogen.

## Ethics Statement

All procedures involving vertebrate animals were carried out according to the Brazilian National Law number 11794 and approved by the Institutional Animal Care and Use Committees from the Faculty of Veterinary Medicine and Zootechnics (protocol number 1423/2008) and the Institute of Biomedical Sciences (protocol number 128/2011), University of São Paulo, São Paulo, Brazil. This study does not involve experimentation with human beings.

## Author Contributions

ACF designed the experiments. LM, CM, MG, and FC generated the biological samples. LM, CM, and MG performed the experiments. JR performed the bioinformatics data analysis. LM, ACF, JR, and AF analyzed the RNA-seq, RNAi, and RT-qPCR data. JR, AF, and LM performed the statistical data analysis. ACF, JR, AF, ML, EE, and SD contributed to reagents, materials, and analysis tools. ACF and LM wrote the manuscript. SD, MG, ML, AF, and JR critically revised the manuscript. All authors read and approved the final manuscript.

## Conflict of Interest Statement

The authors declare that the research was conducted in the absence of any commercial or financial relationships that could be construed as a potential conflict of interest.
