# Supplementary material for: The Transcriptome of the Salivary Glands of Amblyomma aureolatum Reveals the Antimicrobial Peptide Microplusin as an Important Factor for the Tick Protection Against Rickettsia rickettsii Infection
Source: Front Physiol. 2019 May 3;10:529. doi: 10.3389/fphys.2019.00529 (PMC6509419; doi:10.3389/fphys.2019.00529)
Supplement: Supplementary file 1 [file Table_1.DOCX]

**Supplementary Table 1.**

Please access the link below to download the complete dataset of the transcriptome of *A. aureolatum* SG:

<https://s3.amazonaws.com/proj-bip-prod-publicread/transcriptome/Amb_aureolatum/SupplementaryTable1.zip>
