# Supplementary material for: The Transcriptome of the Salivary Glands of Amblyomma aureolatum Reveals the Antimicrobial Peptide Microplusin as an Important Factor for the Tick Protection Against Rickettsia rickettsii Infection
Source: Front Physiol. 2019 May 3;10:529. doi: 10.3389/fphys.2019.00529 (PMC6509419; doi:10.3389/fphys.2019.00529)
Supplement: Supplementary file 2 [file Table_2.DOCX]

Supplementary Table 2. Accession numbers of microplusin sequences used to perform multiple sequence alignment and phylogenetic tree displayed in Figure 2.

| **Species of ticks** | **Acession number** |
| --- | --- |
| *Amblyomma aureolatum* | JAT93257.1 |
| *Amblyomma americanum* | JAG91966.1 |
| *Amblyomma sculptum* | JAC22467.1 |
| *Amblyomma maculatum* | AEO36237.1 |
| *Amblyomma triste* | JAC30433.1 |
| *Hyalomma excavatum* | JAP67221.1 |
| *Ixodes ricinus* | JAA68382.1 |
| *Ornithodoros turicata* | MBY08256.1 |
| *Rhipicephalus appendiculatus* | JAP81743.1 |
| *Rhipicephalus pulchellus* | JAA53972.1 |
| *Rhipicephalus zambeziensis* | MAA15549.1 |
| *Amblyomma hebraeum* | AAR97292.1 |
| *Argas monolakensis* | sp\|Q09JR4.1\|MPSIN_ARGMO |
| *Ixodes scapularis* | XP_002410155.1 |
| *Ornithodoros coriaceus* | ACB70385.1 |
| *Ornithodoros parkeri* | ABR23424.1 |
| *Rhipicephalus microplus* | sp\|Q86LE5.1\|MPSIN_RHIMP |
